# Magnesium Elevation Promotes Neuronal Differentiation While Suppressing Glial Differentiation of Primary Cultured Adult Mouse Neural Progenitor Cells through ERK/CREB Activation

**DOI:** 10.3389/fnins.2017.00087

**Published:** 2017-02-23

**Authors:** Wang Liao, Mujun Jiang, Mei Li, Congli Jin, Songhua Xiao, Shengnuo Fan, Wenli Fang, Yuqiu Zheng, Jun Liu

**Affiliations:** ^1^Department of Neurology, Sun Yat-sen Memorial Hospital, Sun Yat-sen UniversityGuangzhou, China; ^2^Guangdong Province Key Laboratory of Brain Function and Disease, Zhongshan School of Medicine, Sun Yat-sen UniversityGuangzhou, China; ^3^Laboratory of RNA and Major Diseases of Brain and Heart, Sun Yat-sen Memorial Hospital, Sun Yat-sen UniversityGuangzhou, China; ^4^Department of Neurology, Bengbu Medical College, The First Affiliated HospitalBengbu, China; ^5^Department of Neurology, Affiliated Hospital of Guangdong Medical UniversityZhanjiang, China

**Keywords:** adult neural progenitor cells, adult neurogenesis, differentiation, extracellular signal-regulated kinase, fate determination, magnesium

## Abstract

This study aimed to explore the influence of magnesium elevation on fate determination of adult neural progenitor cells (aNPCs) and the underlying mechanism *in vitro*. Adult neurogenesis, which is the generation of functional neurons from neural precursors, occurs throughout life in restricted anatomical regions in mammals. Magnesium is the fourth most abundant ion in mammals, and its elevation in the brain has been shown to enhance memory and synaptic plasticity *in vivo*. However, the effects of magnesium on fate determination of aNPCs, which are vital processes in neurogenesis, remain unknown. NPCs isolated from the dentate gyrus of adult C57/BL6 mice were induced to differentiate in a medium with varying magnesium concentrations (0.6, 0.8, and 1.0 mM) and extracellular signal-regulated kinase (ERK) inhibitor PD0325901. The proportion of cells that differentiated into neurons and glial cells was evaluated using immunofluorescence. Quantitative real-time polymerase chain reaction and Western blot methods were used to determine the expression of β-III tubulin (Tuj1) and glial fibrillary acidic protein (GFAP). The activation of ERK and cAMP response element-binding protein (CREB) was examined by Western blot to reveal the underlying mechanism. Magnesium elevation increased the proportion of Tju1-positive cells and decreased the proportion of GFAP-positive cells. Also, the expression of Tuj1 was upregulated, whereas the expression of GFAP was downregulated. Moreover, magnesium elevation enhanced the activation of both ERK and CREB. Treatment with PD0325901 reversed these effects in a dose-dependent manner. Magnesium elevation promoted neural differentiation while suppressing glial cell differentiation, possibly via ERK-induced CREB activation.

## Introduction

Neurogenesis occurs throughout life in the brain of adult mammals and is restricted mainly to two specific regions of the brain: the subventricular zone (SVZ) of the lateral ventricles and the subgranular zone (SGZ) of the dentate gyrus (DG) of the hippocampus (Abrous and Wojtowicz, 2015). Adult-born neurons can affect the brain function globally in their capacity both as encoding units and as active modifiers of mature neuron firing, synchronization, and network oscillations (Martinez-Marcos et al., 2016). Numerous studies have demonstrated a correlation between the level of hippocampal neurogenesis and cognition, whereas dysfunction of neurogenesis contributes to some pathological processes including epilepsy, Alzheimer's disease, Parkinson's disease, and other degenerative diseases (Kiyota et al., 2015; Hollands et al., 2016).

Adult neurogenesis is a dynamic, finely tuned process that is regulated by various physiological and pathological activities (Egeland et al., 2015; Yang et al., 2015). For instance, cultured hippocampal progenitors increased neuronal differentiation in response to glutamate (Gilley and Kernie, 2011). Growth factors, such as epidermal growth factor (EGF), neurotrophin-derived neurotrophic factor (BDNF), and endothelial growth factor, are also involved in neural stem cell (NSC) maintenance, cell proliferation, and fate specification (Tzeng et al., 2013; Kirby et al., 2015).

Magnesium is the second most abundant intracellular cation after potassium and is involved in more than 600 enzymatic reactions involved in processes including energy metabolism and protein synthesis (de Baaij et al., 2015). Furthermore, magnesium supplements are widely used to treat preeclampsia, depression, coronary artery disease, and asthma (Dribben et al., 2010). Recently, the newly developed compound magnesium L-threonate (mgT), which is capable of elevating magnesium in the brains of mice, was shown to increase synaptic plasticity and enhance learning and memory (Slutsky et al., 2010). The substantial synaptoprotective effects of magnesium elevation in the brain have also been demonstrated in a mouse model of Alzheimer's disease (Li W. et al., 2014). However, the effects of magnesium on neurogenesis remain to be investigated.

The sequential steps of adult neurogenesis include proliferation of NSCs or progenitors, differentiation and fate determination, and survival, maturation, migration, and functional integration into the existing circuitry (Ming and Song, 2011). Newborn NSCs exhibit two basic characteristics: the capacity for self-renewal and differentiation into neurons, astrocytes, and oligodendrocytes (the latter two types are collectively known as glial cells; Zhao et al., 2008; Mu et al., 2010). It was shown that the elevation of magnesium concentration to 2.5 mM above basal levels increased the number of NSCs and some parameters of neurite outgrowth (Vennemeyer et al., 2014). However, the effect of magnesium elevation on fate determination of neuronal cells during neurogenesis remains to be elucidated.

This study indicated that the elevation of magnesium by adding magnesium sulfate (MgSO_4_) or magnesium chloride (MgCl_2_) to the differentiation culture medium increased the expression of βIII-tubulin (Tuj1)-positive cells and decreased the expression of glial fibrillary acidic protein (GFAP)-positive cells after differentiation. These results indicated that magnesium elevation promoted neural differentiation, while suppressing glial differentiation *in vitro*.

Accumulating lines of evidence demonstrate that mitogen-activated protein kinase (MAPK) signaling acts as a rheostat that influences neurogenesis and neural cell fate selection (Li S. et al., 2014; Hosseini Farahabadi et al., 2015). Also, the transcription factor cAMP response element-binding protein (CREB) plays a critical role in memory consolidation via enhanced adult hippocampal neurogenesis (Ortega-Martínez, 2015; Hollands et al., 2016). Other studies showed that endogenous reactive oxygen species regulated neurogenesis in a phosphoinositide 3-OH kinase (PI3K)/Akt-dependent manner (Peltier et al., 2007; Le Belle et al., 2011). This study showed that both the extracellular signal-regulated kinase (ERK) and CREB were activated and might be involved in the underlying mechanism. This was confirmed in experiments using ERK inhibitor PD0325901 and U0126.

This novel study explored the influence of magnesium elevation on fate determination of adult neural progenitor cells (aNPCs) and the underlying mechanism *in vitro* (Bian et al., 2013).

## Materials and methods

### Isolation and culture of adult NPCs

The primary aNPCs were isolated from the DG of 6-week-old male C57BL/6J mice according to a previously described method (Guo et al., 2012). Briefly, the whole brain of adult mice was removed and then sliced into 400 μm sections using an adult mouse matrix (Kent Scientific, CT, USA). The DG was then microdissected from these sections under a microscope and placed in Solution A [30 mM glucose, 26 mM NaCO_3_, 2 mM [4-2-hydroxyethyl)-1-piperazineethanesulfonic acid, pH 7.4 (Thermo Scientific, MA, USA) in Hank's balanced salt solution (Thermo Scientific)] and centrifuged for 10 min at 1000 rpm. The pelleted tissue was dissociated using a MACS Neural Tissue Dissociation Kit for enzymatic digestion (Miltenyi Biotec, CA, USA). The digestion was terminated by adding Dulbecco's modified Eagle's medium [(DMEM)/F-12 medium (Thermo Scientific)] containing 10% fetal bovine serum (FBS, Thermo Scientific). The DG tissue was filtered through a 70 μm cell strainer (Fisher Scientific, MA, USA) and centrifuged for 3 min at 1000 rpm. The pellet was washed with DMEM/F-12 medium supplemented with 10% FBS plus Percoll (GE Healthcare Life Sciences, PA, USA) solution [1:10 Percoll in phosphate-buffered saline (PBS)]. After centrifuging at 1000 rpm for 3 and 15 min, the dissociated cells were resuspended and plated in T25 flasks in proliferation medium [neurobasal medium (Thermo Scientific) with 20 ng/mL EGF (Peprotech), 20 ng/mL basic fibroblast growth factor 2 (Waisman Biomanufacturing), B27 supplement (Thermo Scientific), penicillin-streptomycin (Thermo Scientific), and L-glutamine (Thermo Scientific)] at 37°C under 5% CO_2_. After 7–14 days in culture, the neurospheres were passaged to expand further the number of aNPCs or collected for future experiments. All animal care and experimental procedures used in this study were approved by the Animal Care and Ethics Committee of Sun Yat-sen University, China. The animals were purchased from the Animal Experiment Center of Guangdong Province.

### Differentiation of adult NPCs *in vitro*

For differentiation (Guo et al., 2012), neurospheres from the third passage were collected, dissociated using TrypLE (Life Technologies, USA), and resuspended as a single-cell suspension in magnesium-depleted N2 differentiation medium [500 mL of magnesium-depleted DMEM/F-12 medium (Omega Scientific), 5 mL of N2 (Thermo Scientific), 5 mL of glutamine (Thermo Scientific), and 5 mL of antibiotic–antimycotic (Thermo Scientific)] with a final concentration of 1 μM retinoic acid (Sigma-Aldrich), 1 μM forskolin (Sigma-Aldrich), penicillin–streptomycin, and various concentrations of magnesium (0.6, 0.8, and 1.0 mM). The cells were seeded (5 × 10^4^/cm^2^) in 12-well-cell culture plates coated with poly-L-ornithine (10 μg/mL, Sigma-Aldrich) and laminin (5 μg/mL, BD Biosciences) and differentiated for 6 days. Half of the culture medium was replaced every 2 days, and the cells were harvested at the indicated time points for further analysis. One of the groups was treated with the ERK1/2 inhibitor PD0325901 (Sigma), U0126 (Sigma), for 2 days after seeding (Hosseini Farahabadi et al., 2015). Materials not described were purchased from Thermo-Fisher (PA, USA).

### Magnesium assay

To determine the magnesium content, the culture medium was collected and measured using Calmagite chronometry (BioAssay Systems, CA, USA) according to the manufacturer's protocol (Slutsky et al., 2010). The fluorescent optical density (OD) at 520 nm was used as an indicator of magnesium concentration. All measurements were performed in triplicate in three independent experiments.

### Cell morphology

To evaluate the viability of cells exposed to ERK inhibitors, the cells on the sixth day after differentiation were examined using a light microscope (Zeiss Axiostar Plus, Germany) for any morphological alterations.

### Lactate dehydrogenase cytotoxicity assessment

Lactate dehydrogenase (LDH) is one of the most important oxidative enzymes widely distributed in cell cytoplasm and membranes. Serum LDH activity is often used as an appropriate indicator of cellular damage in cytotoxicity studies (Wu et al., 2015). After differentiation for 6 days, the culture supernatants (*n* = 3 wells) were harvested and the LDH assay was performed using an LDH cytotoxicity detection kit (Roche Diagnostic GmbH, Mannheim, Germany) according to the manufacturer's instructions. Then, 100 μL of cell supernatant was added to 100 μL of LDH substrate buffer. The absorbance was measured at 490 nm after incubating in the dark at room temperature for 20 min, with a reference wavelength of 600 nm, using a computer-controlled microplate reader.

### Immunocytochemistry

Adult NPCs were fixed with 4% paraformaldehyde in 0.2M PBS (7.4) for 20 min at room temperature to identify the adult NPCs and cellular phenotypes after differentiation. The cells were then permeabilized for 10 min with 0.3% Triton X-100 (Sigma-Aldrich) and blocked for 1 h with 10% normal goat serum (Thermo Scientific) in 0.01M PBS. They were then incubated overnight at 4°C with the following primary antibodies: rat anti-mouse nestin (1:100; Millipore, MA, USA), mouse anti-mouse Tuj1 (1:1,000; Millipore), and rabbit anti-mouse GFAP (1:500; Millipore). After three washes with PBS, the samples were incubated in tetramethylrhodamine isothiocyanate-conjugated goat anti-rabbit or rabbit anti-mouse secondary antibody (1:100; ZSGB-BIO, Beijing, China) for 1 h to immunolabel the anti-Tuj1 and anti-GFAP antibodies, respectively. The nuclei were counterstained with 4′,6-diamidino-2-phenylindole (DAPI; 1 μg/mL; Sigma-Aldrich) for 5 min. The samples were subsequently washed with PBS three times prior to observation. Fluorescence images were obtained by the Nikon ECLIPSE Ti fluorescence microscope (Nikon Corporation, Tokyo, Japan) using NIS-Elements BR 3.0 software (Nikon Corporation, Tokyo, Japan).

### Cell counting

Fluorescent images of immunopositive cells were captured as described for immunocytochemistry (ICC). The immunopositive ratios for each treatment condition after differentiation were calculated using Image-ProPlus6.0 software (Media Cybernetics, MD, USA) by counting the number of immunopositive cells (immuno-labeled using the neuronal or astrocytic markers) divided by the total number of cells (all cells stained with DAPI); 10 random fields were counted from three independent experiments (Chu et al., 2015; Kim et al., 2015).

### RNA extraction and quantitative real-time polymerase chain reaction

Total RNA was isolated from differentiated aNPCs for each treatment condition using an RNA Purification Kit according to the manufacturer's instructions (Thermo Scientific). Subsequently, cDNA was synthesized from 2 μg of purified total RNA using a First Strand cDNA Synthesis Kit (Thermo Scientific). Quantitative real-time polymerase chain reaction (RT-PCR) was performed using an MyiQ2 real-time PCR Detection System (Bio-Rad) with the SYBR Premix Ex Taq (TaKaRa, Tokyo, Japan). qRT-PCR was initiated using an activation step at 95°C for 15 min, followed by 40 amplification cycles of denaturation at 95°C for 10 s, annealing at 55°C for 30 s, and extension at 72°C for 30 s. Glyceraldehyde-3-phosphate dehydrogenase (GAPDH) served as the internal control. The sequences of the forward and reverse primers used to detect the expression levels of the genes of interest were as follows: GFAP: 5′-AGCT ACA TCG AGA AGG TCC GC-3′, 5′-GTC TCT TGCATG TTA CTG GTG-3′; Tuj1: 5′-TAGACCCCAGCGGCAACTAT-3′ and 5′-GTTCCAGGTTCCAAGTCCACC-3′; GAPDH: 5′-ATCTTCTTGTGCAGT GCCAG-3′ and 5′-CGTTGA TGGCAA CAA TCT CC-3′. All measurements were performed in triplicate in three independent experiments. The relative changes in gene expression levels were presented as values of 2^Ct (GAPDH) −Ct(gene of interest)^. Relative expression levels were analyzed using the 2^−ΔΔCT^ method, as described previously (Liu et al., 2009).

### Western blot analysis

The primary aNPCs were harvested after differentiation for Western blot analysis, and equal amounts of proteins (Bicinchoninic Acid Protein Assay Kit) were separated by 4–20% polyacrylamide gel electrophoresis as described previously (Zhao et al., 2013). Briefly, the cell culture medium was removed, and the cells were washed twice with 0.01M PBS precooled to 4°C. The cells were then lysed with appropriate amounts of boiling denaturing lysate buffer (1% sodium dodecyl sulfate, 1 mM sodium orthovanadate, 10 mM Tris-HCl, pH 7.4) supplemented with a protease inhibitor cocktail (Roche Diagnostics, IN, USA). Proteins were transferred onto nitrocellulose membranes after quantification and incubated overnight at 4°C with various primary antibodies in blocking solutions: GAPDH (1:5000), CREB (1:1000), phospho-CREB (Ser133; 1:2000), phospho-p44/42 MAPK, and total p44/42 MAPK, Akt (1:1000), phospho-Akt (Ser473; 1:500), phospho-PI3 kinase p85 (Tyr458)/p55 (Tyr199; 1:1000), PI3 kinase p110α (1:1000), GFAP (1:1000), and Tuj1 (1:1000). Primary antibodies were all purchased from Cell Signaling Technology (MA, USA) except those specific for GFAP and Tuj1, which were purchased from Millipore (MA, USA). The membranes were washed with Tris-buffered saline and Tween 20, incubated for 1 h with horseradish peroxidase (HRP)-conjugated goat anti-rabbit IgG(H+L) or HRP-conjugated goat anti-mouse IgG (H+L) secondary antibodies (1: 20,000; ZSGB-BIO), and visualized using an enhanced chemiluminescence (GE Healthcare, WI, USA) detection kit. Immunoreactivity was visualized by exposure to an x-ray film. The relative densities of bands were analyzed using a gel imaging analysis system (Genetics Inc., USA). Each experiment was performed at least three times, and representative blots are presented.

### Statistical analysis

Quantitative data are expressed as mean ± standard deviation and analyzed using one-way analysis of variance followed by Bonferroni *post-hoc* mean comparisons using SPSS 20.0 (SPSS Inc., IL, USA). A *P* < 0.05 was considered to indicate statistical significance.

## Results

### Adult NPCs were maintained as neurospheres *in vitro* and retained their stem cell characteristics

**Figure 1:**
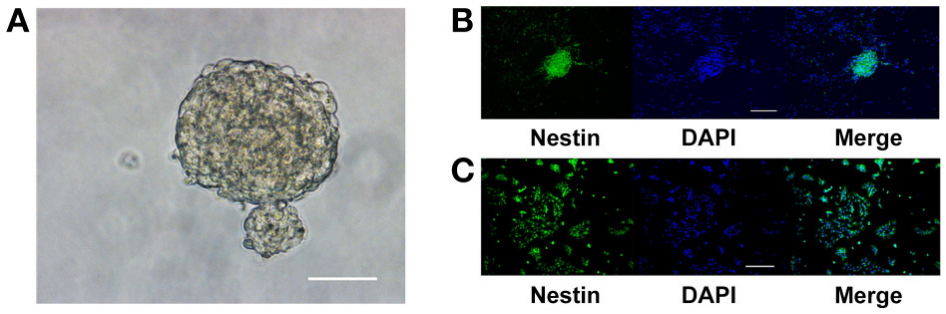
**Adult NPCs were maintained as neurospheres *in vitro*, and their stem cell characteristics were retained. (A)** NPCs isolated from the hippocampus of adult C57BL/6J mice aggregated as neurospheres, scale bar = 50 μm. **(B)** The neurospheres continue to express the stem cell marker, nestin, after three passages, scale bar = 100 μm. **(C)** Upon dissociation and seeding as a monolayer in the growth media, the ratio of nestin-positive cells in the culture was (93.46 ± 2.54)%, scale bar = 400 μm. NPCs, Neural progenitor cells.

NPCs isolated from the hippocampus of adult C57BL/6J mice aggregated as neurospheres in NSC culture medium (Figure 1A). The neurospheres continued to express nestin, indicating that the cells were either NSCs or type 2 progenitor cells, after three passages (Figure 1B). Upon dissociation and seeding as a monolayer in growth media, the percentage of nestin-positive cells in the culture was (93.46 ± 2.54)% (Figure 1C). Thus, these aNPCs were an appropriate *in vitro* model for studying the effects of magnesium on the stem/progenitor pool in the hippocampus because these cells proliferated and retained their stem cell characteristics after multiple passages.

### Cell morphology and LDH activity were not changed at proper magnesium and ERK inhibitor concentrations

**Figure 2:**
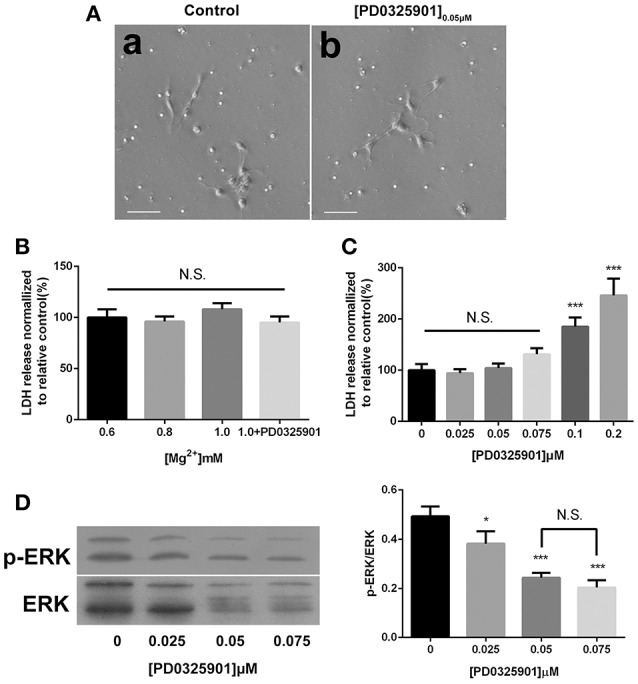
**Effect of different culture conditions on cell morphology, LDH release, and ERK activation. (A)** Cell morphology was not changed after differentiation in the presence of ERK inhibitor PD0325901 (0.05 μM) compared with the control group, scale bar = 50 μm. (a) Control; (b) PD0325901 (0.05μM). **(B)** LDH releases were not changed at various magnesium concentrations (0.6, 0.8, and 1.0 mM) and in the presence of 0.05 μM PD0325901 (*P* > 0.05; *n* = 3). N.S., *P* > 0.05. **(C)** LDH release increased with the increase in PD0325901 concentration to 0.1 μM (*P* < 0.001; *n* = 3). N.S., *P* > 0.05, ^***^P < 0.001 vs. [PD0325901]_0_μM. **(D)** p-ERK/ERK was reduced on exposure to PD0325901 at a concentration of 0.05 μM compared with the control group. No significant change was observed between 0.05 and 0.075 μM (*P* > 0.05; *n* = 3). (a) 0μM; (b) 0.025μM; (c) 0.05μM; (d) 0.075μM. N.S., *P* > 0.05, ^*^P < 0.05 vs. [PD0325901]_0_μM, ^***^P < 0.001 vs. [PD0325901]_0_μM. ERK, Extracellular signal–regulated kinase; LDH, lactate dehydrogenase.

The light microscopic examination was performed to estimate the viability of the cells exposed to ERK inhibitor PD0325901. No morphological change was found at a concentration of 0.05 μM after 6 days of differentiation (Figure 2A). The culture supernatants were harvested after culturing for 6 days, and the amount of LDH leakage into the medium was measured to assess the cytotoxicity of various magnesium concentrations in the presence of PD0325901. Figure 2B shows that LDH activity did not significantly increase at the experimental magnesium concentrations (0.6, 0.8, and 1.0 mM) and in the presence of ERK inhibitor PD0325901 (0.05 μM; *P* > 0.05). However, LDH release was significantly elevated with the increase in PD0325901 concentration to 0.1 and 0.2 μM (*P* < 0.001; Figure 2C).

### Percentage of Tuj1-positive increased and the percentage of GFAP-positive cells decreased after differentiation under conditions of elevated magnesium

**Figure 3:**
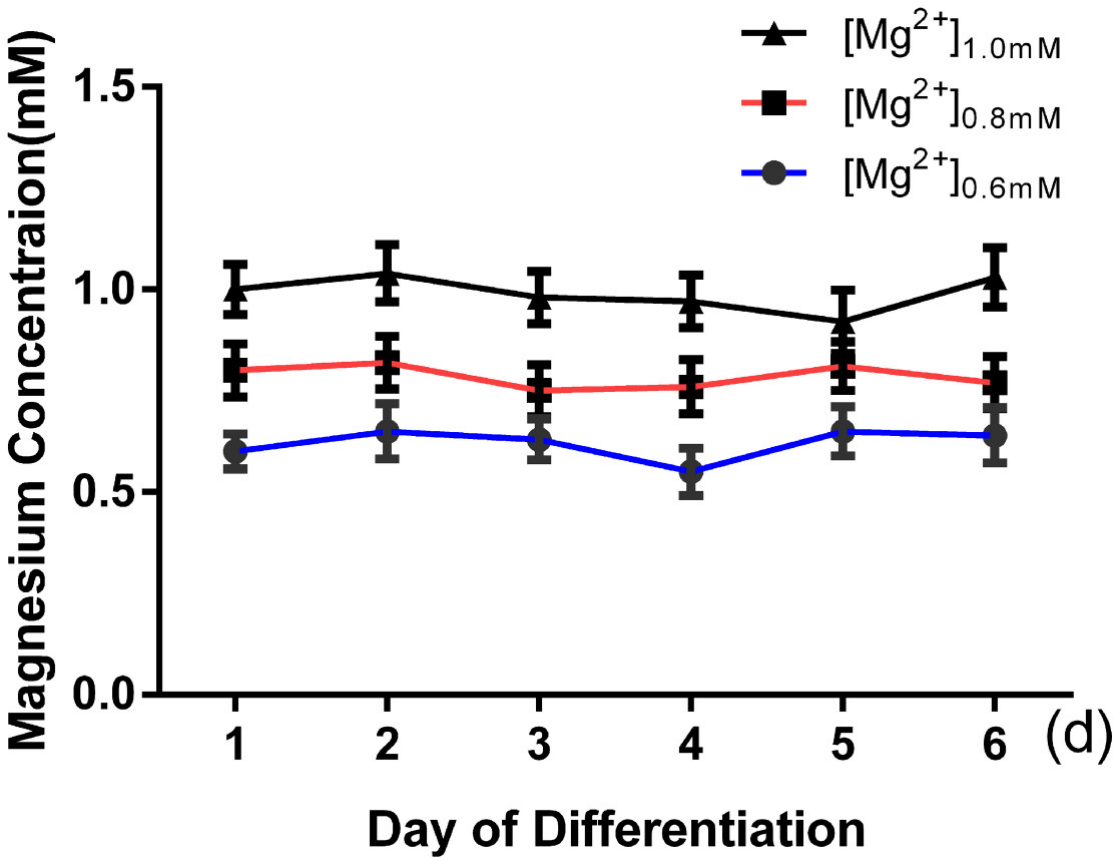
**Magnesium concentrations in the culture medium were stable during differentiation**. The magnesium concentrations were not significantly changed with time in each group (*P* > 0.05; *n* = 3).

**Figure 4:**
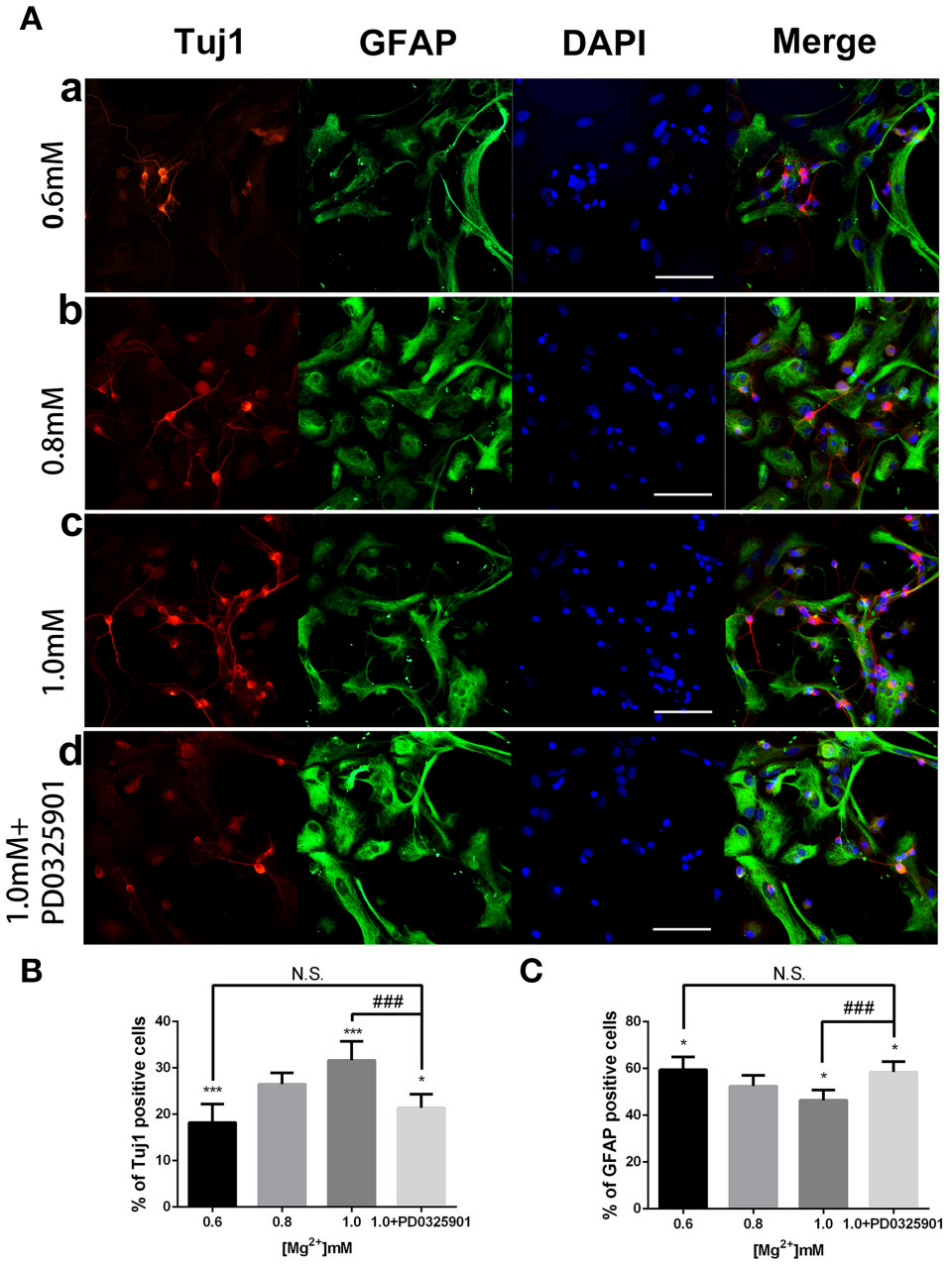
**Immunocytochemistry of adult NPCs differentiated at various magnesium concentrations and in the presence of an ERK inhibitor**. **(A)** The percentage of Tuj1-positive cells increased **(B)** and the percentage of GFAP-positive cells decreased **(C)** after differentiation with an increase in magnesium concentration (*P* < 0.05). Supplemented with PD0325901 (0.05 μM), the percentage of Tuj1-positive cells decreased and the percentage of GFAP-positive cells increased compared with the control group (0.8 mM) and the group with an elevated magnesium concentration (1.0 mM; *P* < 0.05), but no significant change was observed compared with the group with low magnesium concentration (0.6 mM; *P* > 0.05; *n* = 10). Scale bar = 100 μm. (a) 0.6 mM; (b) 0.8 mM; (c) 1.0 mM; (d) 1.0 mM + PD0325901. ^*^P < 0.05 vs. [Mg^2+^]_0.8mM_, ^***^P < 0.001 vs. [Mg^2+^]_0.8mM_; N.S., *P* > 0.05 vs. [Mg^2+^]_0.6mM_; ^###^*P* < 0.001 vs. [Mg^2+^]_1.0mM_. ERK, extracellular signal-regulated kinase; GFAP, glial fibrillary acidic protein; NPC, neural progenitor cell.

ICC was performed after 6 days of differentiation in a medium containing various concentrations of magnesium to determine the effect of magnesium on fate determination of aNPCs. Magnesium concentrations in the culture medium were stable during differentiation according to the results of magnesium assays (*P* > 0.05; Figure 3). NPCs differentiated mainly into neurons and glia. Thus, the neuronal marker Tuj1 was used to identify neurons, and GFAP was used as the specific indicator of glia. Ten random fields were chosen from three independent experiments per treatment, and the average number of positive cells was calculated. Each calculation was performed by two experimenters independently without prior knowledge of the label. Treatment with elevated magnesium (1.0 mM) was found to significantly increase the percentage of Tuj1-positive cells (*P* < 0.001) compared with the control group (0.8 mM). Conversely, the percentage of GFAP^+^ cells decreased (*P* < 0.05) compared with the control group (Figure 4). Moreover, significant changes were also observed at magnesium concentrations of 0.6 and 0.8 mM for both Tuj1- and GFAP-positive cells (*P* < 0.05). The total cell number was found to be unchanged on counting the DAPI-positive cells. Also, long neurites could be identified in Tuj1-positive cells at all magnesium concentrations after 6 days of differentiation (Figure 4).

### Expression of Tuj1 was upregulated and the expression of GFAP was downregulated under conditions of elevated magnesium

**Figure 5:**
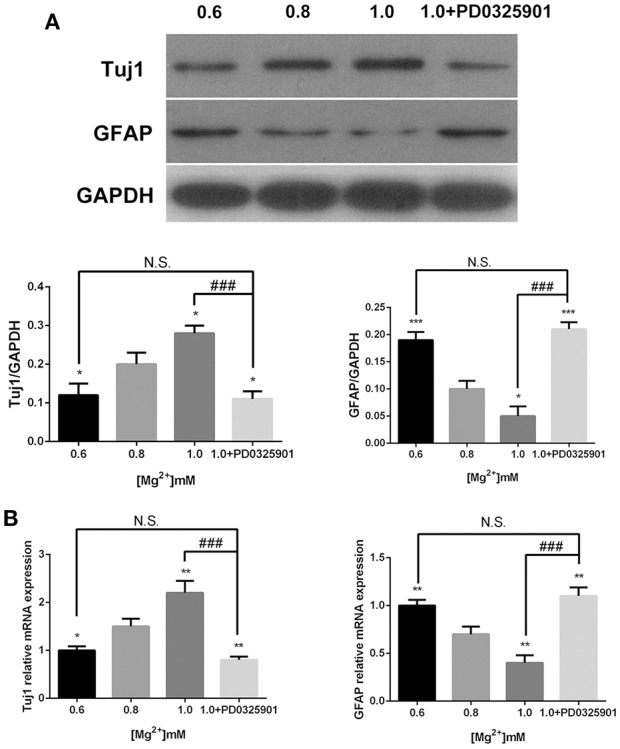
**Expression of Tuj1 and GFAP at elevated magnesium and ERK inhibitor PD0325901**. The expression of Tuj1 was upregulated and the expression of GFAP was downregulated after differentiation with an increase in magnesium concentration. Supplemented with PD0325901 (0.05 μM), the expression of Tuj1 was downregulated and the expression of GFAP was upregulated compared with the control group (0.8 mM) and the group with elevated magnesium concentration (1.0 mM; *P* < 0.05), but no significant change was observed compared with the group with low magnesium concentration (0.6 mM; *P* > 0.05; *n* = 3). **(A)** Western blot analysis of the expression of Tuj1 and GFAP. **(B)** qRT-PCR analysis of the relative mRNA expression of Tuj1 and GFAP. ^*^P < 0.05 vs. [Mg^2+^]_0.8mM_, ^**^P < 0.01 vs. [Mg^2+^]_0.8mM_, ^***^P < 0.001 vs. [Mg^2+^]_0.8mM_; N.S., *P* > 0.05 vs. [Mg^2+^]_0.6mM_; ^###^*P* < 0.001 vs. [Mg^2+^]_1.0mM_. GFAP, glial fibrillary acidic protein; qRT-PCR, quantitative real-time polymerase chain reaction.

In accordance with the ICC results, the Western blot analysis showed that the expression of Tuj1 increased (*P* < 0.05) when GFAP expression decreased (*P* < 0.05) at various magnesium concentrations (Figure 5A). The qRT-PCR analysis showed that magnesium elevation increased the expression level of Tuj1 mRNA (*P* < 0.05) and decreased the expression level of GFAP mRNA (*P* < 0.01; Figure 5B). Significant changes in the expression of these markers were also observed at magnesium concentrations of 0.6 and 0.8 mM (*P* < 0.05). These results indicated that magnesium elevation influenced fate determination of NPCs by promoting neural differentiation and inhibiting glial differentiation.

### MgCl_2_ had the same effect on fate determination of NPCs as that of MgSO_4_

**Figure 6:**
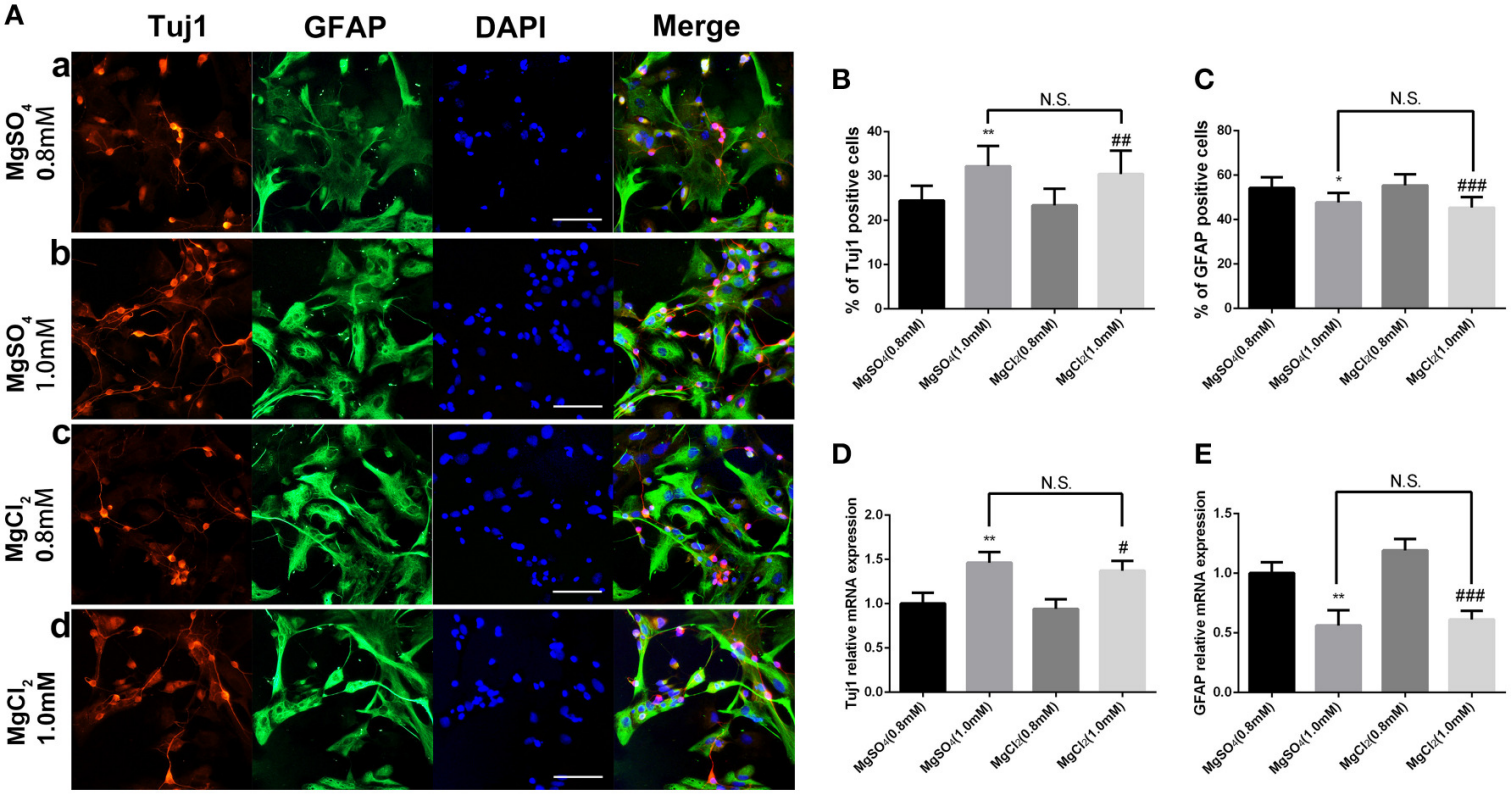
**Effect of MgCl_2_ and MgSO_4_on the expression of Tuj1 and GFAP**. The expression of Tuj1 was upregulated and the expression of GFAP was downregulated after differentiation with both magnesium solutions (*P* < 0.05). No significant change was observed between MgCl_2_and MgSO_4_ treatment (*P* > 0.05). **(A–C)** The percentage of Tuj1-positive cells increased and the percentage of GFAP-positive cells decreased (*n* = 10). **(D,E)** The qRT-PCR analysis of the relative mRNA expression of Tuj1 and GFAP (*n* = 3). N.S., *P* > 0.05, ^*^P < 0.05 vs. [MgSO_4_]_0.8mM_, ^**^P < 0.01 vs. [MgSO_4_]_0.8mM_; #*P* < 0.05 vs. [MgCl_2_]_0.8mM_, ^##^*P* < 0.01 vs. [MgCl_2_]_0.8mM_, ^###^*P* < 0.001 vs. [MgCl_2_]_0.8mM_. GFAP, glial fibrillary acidic protein; qRT-PCR, quantitative real-time polymerase chain reaction.

MgCl_2_ (0.8 and 1.0 mM) solution was added to aNPCs to determine whether the anion or sulfate influenced response. Figure 6 shows that exposure to both magnesium solutions resulted in an increased proportion of Tuj1-positive cells (*P* < 0.01) and a decreased proportion of GFAP-positive cells (*P* < 0.001). No significant difference was found between MgCl_2_ and MgSO_4_ treatment (*P* > 0.05; Figure 6). In accordance with the ICC results, the qRT-PCR analysis showed that MgCl_2_ (1.0 mM) increased the expression level of Tuj1 mRNA (*P* < 0.05) and decreased the expression level of GFAP mRNA (*P* < 0.001). There was no significant change between MgCl_2_ and MgSO_4_ group (*P* > 0.05). These results indicated that MgCl_2_ had the same effect on fate determination of NPCs as that of MgSO_4._

### ERK/CREB activation was necessary for the effect of magnesium elevation on fate determination of NPCs

**Figure 7:**
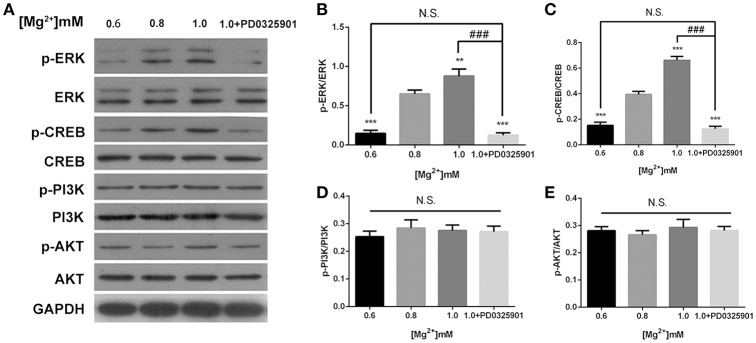
**ERK/CREB and PI3K/Akt activation in response to elevated magnesium and ERK inhibitor PD0325901**. **(A)** Both p-ERK/ERK and p-CREB/CREB ratios increased with the elevation of magnesium (*P* < 0.01) **(B,C)**. Magnesium elevation has no significant effect on p-PI3K/PI3K and p-Akt/Akt ratios (*P* > 0.05) **(D,E)**. **(A-E)** Supplemented with PD0325901 (0.05 μM), both p-ERK/ERK and p-CREB/CREB ratios decreased compared with the control group (0.8 mM) and the group with elevated magnesium concentration (1.0 mM) without PD0325901 (*P* < 0.05), but no significant change was observed compared with the group with low magnesium concentration (0.6mM; *P* > 0.05; *n* = 3). ^**^P < 0.01 vs. [Mg^2+^]_0.8mM_; ^***^P < 0.001 vs. [Mg^2+^]_0.8mM_; N.S., *P* > 0.05 vs. [Mg^2+^]_0.6mM_; ^###^*P* < 0.001 vs. [Mg^2+^]_1.0mM_. CREB, cAMP response element-binding protein; ERK, extracellular signal-regulated kinase.

The effects of magnesium elevation on the ERK/CREB and PI3K/Akt pathways were investigated to elucidate the mechanisms underlying the effect of magnesium on fate determination of NPCs. The p-ERK/ERK and p-CREB/CREB ratios increased with the elevation of magnesium concentration (*P* < 0.01). However, the elevation of magnesium concentration had no significant effect on either PI3K or Akt (*P* > 0.05; Figure 7).

The modulatory effect of ERK inhibitor PD0325901 on ERK1/2 activation in differentiated adult NPCs was investigated to clarify further the relationship between the ERK/CREB pathway and fate determination of cells. As shown in Figure 2C, LDH releases were not significantly changed at a PD0325901 concentration below 0.075μM. Furthermore, ERK activity could be efficaciously inhibited compared with the control when exposed to PD0325901 at a concentration above 0.025 μM (Figure 2D). Also, no significant change in the p-ERK/ERK ratio was observed at concentrations of 0.05 and 0.075 μM. As a result, 0.05 μM was chosen as the working concentration for further experiments. When supplemented with PD0325901 (0.05 μM), no significant change in the percentages of Tuj1- and GFAP-positive cells was observed at an elevated magnesium concentration (1.0 mM) compared with those at a low magnesium concentration (0.6 mM; *P* > 0.05; Figure 4). Consistent with these results, the Western blot analysis (Figure 5A) and RT-PCR (Figure 5B) showed that the expression of Tuj1 and GFAP was not significantly changed compared with the expression at low magnesium concentration (*P* > 0.05). Supplemented with another MEK inhibitor U0126 (0.3 μM; Huang et al., 2017), both p-ERK/ERK and p-CREB/CREB ratios decreased compared with the group with elevated magnesium (1.0 mM) without U0126 (*P* < 0.01). The percentage of Tuj1-positive cells decreased and the percentage of GFAP-positive cells increased compared with the group with elevated magnesium concentration without U0126 (*P* < 0.01; Supplementary Figure 2).

**Figure 8:**
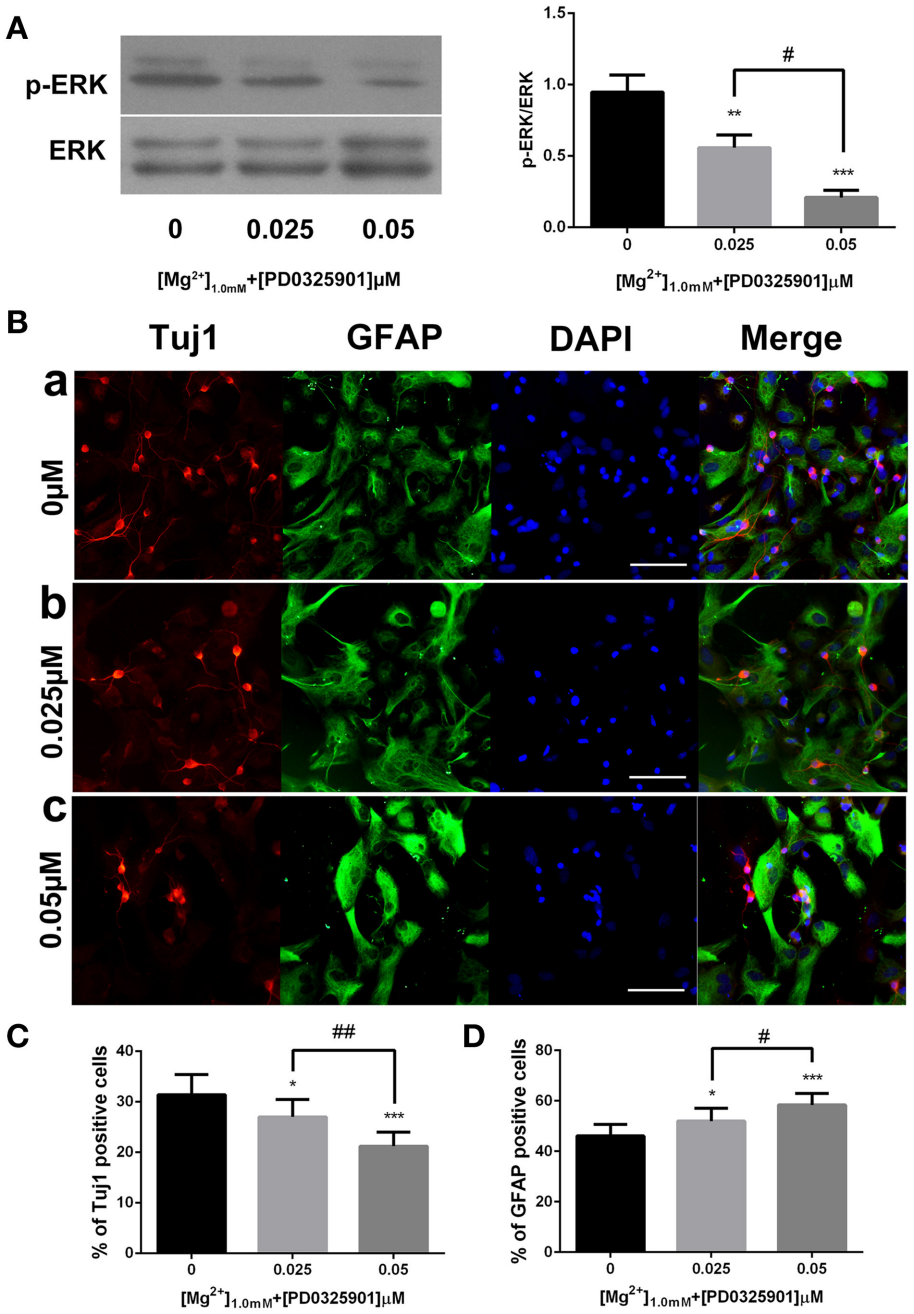
**ERK activation and proportion of Tuj1^+^ and GFAP^+^ cells at various PD0325901 concentrations with magnesium elevation. (A)** On exposure to 0.025 and 0.05 μM PD0325901, the p-ERK/ERK ratio at 1.0 mM magnesium concentration significantly reduced compared with the control group (0 μM; *P* < 0.01). Moreover, the change was significant between 0.025 and 0.05 μM (*P* < 0.05; *n* = 3). (a) 0μM; (b) 0.025μM; (c) 0.05μM. **(B–D)** Percentage of Tuj1-positive cells increased and the percentage of GFAP-positive cells decreased at PD0325901 concentrations of 0.025 and 0.05 μM compared with the control group (0 μM; *P* < 0.05). A significant change was also observed between 0.025 and 0.05 μM (*P* < 0.05; *n* = 10), scale bar = 100 μ m. (a) 0 μM; (b) 0.025 μM; (c) 0.05 μM. ^*^P < 0.05 vs. [PD0325901]_0μM_, ^**^P < 0.01 vs. [PD0325901]_0_μM, ^***^P < 0.001 vs. [PD0325901]_0μM_, ^#^*P* < 0.05 vs. [PD0325901]_0μM_, ^##^*P* < 0.01 vs. [PD0325901]_0.025μM_.

The dose-dependent effect of PD0325901 was investigated to demonstrate further the effect of ERK activation on the influence of magnesium elevation on fate determination. On exposure to 0.025 and 0.05 μM PD0325901, the p-ERK/ERK ratio at 1.0 mM magnesium significantly reduced compared with the control group (0 μM; *P* < 0.01). Moreover, the change was significant between 0.025 and 0.05 μM (*P* < 0.05). Consistent with this, the percentage of Tuj1-positive cells increased, and the percentage of GFAP-positive cells decreased at PD0325901 concentrations of 0.025 and 0.05 μM compared with the control group (0 μM; *P* < 0.05). A significant change was observed between 0.025 and 0.05 μM (*P* < 0.05; Figure 8B). Altogether, Figure 8 shows that ERK activation and promotion of neuronal differentiation induced by magnesium elevation (1.0 mM) could be inhibited by PD0325901 in a dose-dependent manner.

## Discussion

Adult neurogenesis is the process of generation of neurons (Tiwari et al., 2015). While much has been learned about the molecular regulators of different aspects of adult neurogenesis, their effects on fate determination of neuronal vs. glial cells is a current focus of research. The anatomical microenvironment that surrounds stem cells and functions to control their development *in vivo* is defined as the neurogenic niche (Heng et al., 2015). The effect of magnesium concentration in the neurogenic niche on the process of fate determination of NPCs remains largely unexplored.

The elevation of magnesium level in the brain by a newly developed magnesium compound (MgT) has been demonstrated to enhance both short-term synaptic facilitation and long-term potentiation, thereby improving learning and memory functions (Slutsky et al., 2010). The present study investigated the influence of magnesium on the differentiation fate of NPCs and its underlying mechanism.

The normal magnesium concentration in human cerebrospinal fluid is reported to be 0.66 ± 0.14 mM (Sakamoto et al., 2005). The differentiation of adult NPCs was induced in a culture medium containing various concentrations of magnesium (0.6, 0.8, and 1.0 mM) in this study. The elevation of magnesium concentration from 0.8 to 1.2 mM for up to 2 weeks resulted in an increased synaptic number and had no obvious toxic effects (Slutsky et al., 2004). In accordance with other researches, 1.0 mM magnesium was shown to have no effect on cell viability in the present study (Figure 2B). Thus, 1.0 mM magnesium represented an appropriate concentration for use in experiments to investigate the effects of magnesium on neuronal fate.

The present data indicated that the percentages of Tuj1-positive cells increased and GFAP-positive cells decreased after differentiation at elevated magnesium concentrations (Figure 4). Consistent with this, the expression of Tuj1 was upregulated while the expression of GFAP was downregulated with the elevated magnesium concentration (Figure 5). There was no significant change between MgCl_2_ and MgSO_4_ group (Figure 6). This led to the conclusion that magnesium elevation promoted neuronal stem cells to differentiate into neurons while inhibiting glial differentiation. Vennemeyer et al. demonstrated that some parameters of neurite outgrowth increased with elevated magnesium when NSCs were induced to differentiate into neurons on uncoated plastic, while NSC differentiation into neurons was not altered by either substrate changes or magnesium supplementation (Vennemeyer et al., 2014). This study clearly indicated that magnesium could affect the NSC differentiation, although this was not consistent with the results of the present study. First, it can be speculated that this discrepancy might be accounted for by the complexity of the regulation of fate determination of NSCs by external stimuli, and that the effects depended on timing, dose/duration, specific paradigms, models, and methods of analysis. Second, it is also proposed that the difference in characteristics of aNPCs isolated from adult mice and other NSCs might account for the aforementioned difference in results. Third, it might be that the cells were plated on laminin and did not show any response to increasing magnesium concentrations while neurosphere cells were plated on both polyornithine and laminin.

According to the present data, ERK/CREB activation was enhanced by an increase in magnesium concentration and reversed by ERK inhibitor to the level at low magnesium concentration (Figure 7). Accordingly, the expression of Tuj1 and GFAP was also reversed to the expression at low magnesium concentration (Figures 4, 5), indicating that magnesium elevation regulated adult NPC differentiation via the ERK/CREB pathway. Moreover, the activation of ERK by magnesium elevation could be inhibited by PD0325901 in a dose-dependent manner (Figure 8A). Also, the percentage of Tuj1-positive cells increased, and the percentage of GFAP-positive cells decreased in the presence of PD0325901 in a dose-dependent manner (Figure 8B). The role of ERK/CREB activation in the process of fate determination of NPCs was also confirmed by another MEK inhibitor, U0126 (0.3 μM; Supplementary Figure 2).

This was consistent with the study by Wang et al. demonstrating the importance of the MAPK signaling pathway in regulating adult neurogenesis. Furthermore, the conditional activation of endogenous ERK was sufficient to enhance adult neurogenesis, thereby improving the olfactory function both under normal conditions and after injury (Wang et al., 2015). The results of the present study were also consistent with the findings of Lee et al., which indicated that BDNF increased neurogenesis in the hippocampus by triggering ERK1/2 activation, which sequentially activated CREB (Lim et al., 2014). Also, Peltier and colleagues used adult NPCs to demonstrate that PI3K/Akt signaling integrated extracellular signaling information to promote cellular proliferation and inhibit differentiation in adult neural progenitors (Peltier et al., 2007). This was in contrast to the results of the present study, which did not reveal any effects of elevated magnesium on the PI3K/Akt pathway (Figure 7; Miyashita et al., 2012).

The mechanism of how magnesium influences the ERK/CREB pathway remains to be explored. MEK1/2 phosphorylates and activates ERK1/2 via the phosphorylation of the Thr and Tyr residues within their activation loop. The extent and duration of the phosphorylation of ERK1/2 are regulated by two different mechanisms (Rubinfeld and Seger, 2005): first, the regulation of the interaction between MEK1/2 and ERK1/2 and second, the regulation of protein phosphatases including Thr phosphatases, Tyr phosphatases, and MAP kinase phosphatases. Magnesium might regulate ERK activity by affecting the binding of MEK1 to ERK1/2. It was demonstrated that magnesium deprivation decreased ERK activity and re-addition of magnesium reversed the effect. Glutathione-S-transferase pull-down and coimmunoprecipitation assays showed that CA-MEK1 and DN-MEK1 bound to ERK1/2 in the presence of magnesium. These results indicated that the MEK-ERK cascade was regulated by increased levels of p-ERK1/2 induced by magnesium (Ikari et al., 2010a,b). However, this effect was demonstrated using a renal epithelial cell line. Further studies are required to validate this effect in NPCs. Bizen et al. proposed that magnesium deprivation attenuates the function of protein phosphatase 2A (PP2A), which downregulates the phosphorylation of MEK1/2(Bae and Ceryak, 2009; Bizen et al., 2014). Magnesium deprivation may increase the level of p-MEK1 mediated by the inhibition of PP2A. However, the effect of magnesium elevation on NPC needs investigation. An essential catalytic step for MAP kinase involves the binding of an ATP molecule to its active site. It was demonstrated that ATP was bound in the cleft between the N-terminal and C-terminal domains of ERK2, and this process was mediated by magnesium ions (Zhang et al., 2012). However, no ERK phosphorylation is involved in this process.

It has also been postulated that magnesium elevation depolarizes the action potential threshold, decreasing the number of action potentials and depolarizing the neuronal resting potential in a concentration-dependent manner (Dribben et al., 2010). aNPCs were treated with 15 mM KCl, which was reported to be a depolarizing concentration (Jhaveri et al., 2015). However, no significant change in the expression of Tuj1 or GFAP was found (Supplementary Figure 1). Dribben et al. showed that an increase in magnesium concentration from 1 to 10 mM shifted Na^+^ channel activation by ~ +15 mV and depolarized the resting membrane potential by 5.2 mV (Dribben et al., 2010). It was supposed that a rise in magnesium concentration from 0.8 to 1 mM might not be enough to cause any effect on the potential action or membrane potential. However, the role of potential action in the process of fate determination of NPCs cannot be excluded yet. Further studies are required to clarify this issue. What is more, other divalent metal ions (like Zn^2+^) should be explored to rule out the effect of non-specific electrostatic or ionic strength on fate determination of NPCs.

In conclusion, the present study indicated that magnesium elevation promoted neuronal differentiation while suppressing glial differentiation of aNPCs through ERK/CREB activation. Further investigation is required to comfirm this effect *in vivo*. The results of the present study might provide an improved understanding of the effects of magnesium elevation on fate determination of aNPCs, which are vital processes in neurogenesis. Moreover, these mechanistic studies might provide the basis for new strategies to enhance functional neurogenesis in regenerative medicine.

## Ethics statement

This study was carried out in accordance with the recommendations of the Animal Care and Ethics Committee of Sun Yat-sen University, China. The protocol was approved by the Animal Care and Ethics Committee of Sun Yat-sen University, China.

## Author contributions

WL and MJ participated in the design of the study and drafted the manuscript. ML performed the statistical analysis. CJ and SX carried out immunofluorescent staining. WF and SF performed RT-PCR and cell count. YZ performed Western blot. JL designed the experiments and helped draft the manuscript. All authors read and approved the final manuscript.

## Funding

This study was supported by grants to JL from the National Nature Science Foundation of China (No. 81372919), the Natural Science Foundation of Guangdong Province, China (No. 2014A030313016), and the Science and Technology Planning Project of Guangdong Province, China (No. 2013B021800098).

### Conflict of interest statement

The authors declare that the research was conducted in the absence of any commercial or financial relationships that could be construed as a potential conflict of interest.
