# Supplementary material for: Magnesium Elevation Promotes Neuronal Differentiation While Suppressing Glial Differentiation of Primary Cultured Adult Mouse Neural Progenitor Cells through ERK/CREB Activation
Source: Front Neurosci. 2017 Feb 23;11:87. doi: 10.3389/fnins.2017.00087 (PMC5322230; doi:10.3389/fnins.2017.00087)
Supplement: Supplementary file 1 [file DataSheet1.DOCX]

Supplementary Material

**Magnesium elevation promotes neuronal differentiation while suppressing glial differentiation of primary cultured adult mouse neural progenitor cells through ERK/CREB activation**

**Running title:** Magnesium elevation promotes neuronal differentiation

Wang Liao^1,4,5,†^, Mujun Jiang^2,†^, Mei Li^1^, Congli Jin^3^, Songhua Xiao^1^, Shengnuo Fan^1^, Wenli Fang^1^, Yuqiu Zheng^1^, Jun Liu^1,4,5,*^

^1^Department of Neurology, Sun Yat-Sen Memorial Hospital, Sun Yat-Sen University, Guangzhou ,Guangdong 510120, China

^2^Department of Neurology, The First Affiliated Hospital, Bengbu Medical College, Bengbu, Anhui 233004, China

^3^Department of Neurology, Affiliated Hospital of Guangdong Medical University, Zhanjiang, Guangdong 524000, China

^4^Guangdong Province Key Laboratory of Brain Function and Disease, Zhongshan School of Medicine, Sun Yat-sen University, Guangzhou ,Guangdong 510080, China

^5^Laboratory of RNA and Major Diseases of Brain and Heart, Sun Yat-sen Memorial Hospital, Sun Yat-sen University, Guangzhou 510120, China

^†^The authors Wang Liao and Mujun Jiang contributed equally to this work.

**^*^Corresponding Author:**

Jun Liu

Department of Neurology, Sun Yat-sen Memorial Hospital, Sun Yat-sen University, 107 Yanjiang West Road, Guangzhou 510120, China

<Tel:86-20-81332621>

Fax number: 86-20-81332620

E-mail: [docliujun@hotmail.com](mailto:docliujun@hotmail.com)

Supplementary Figure


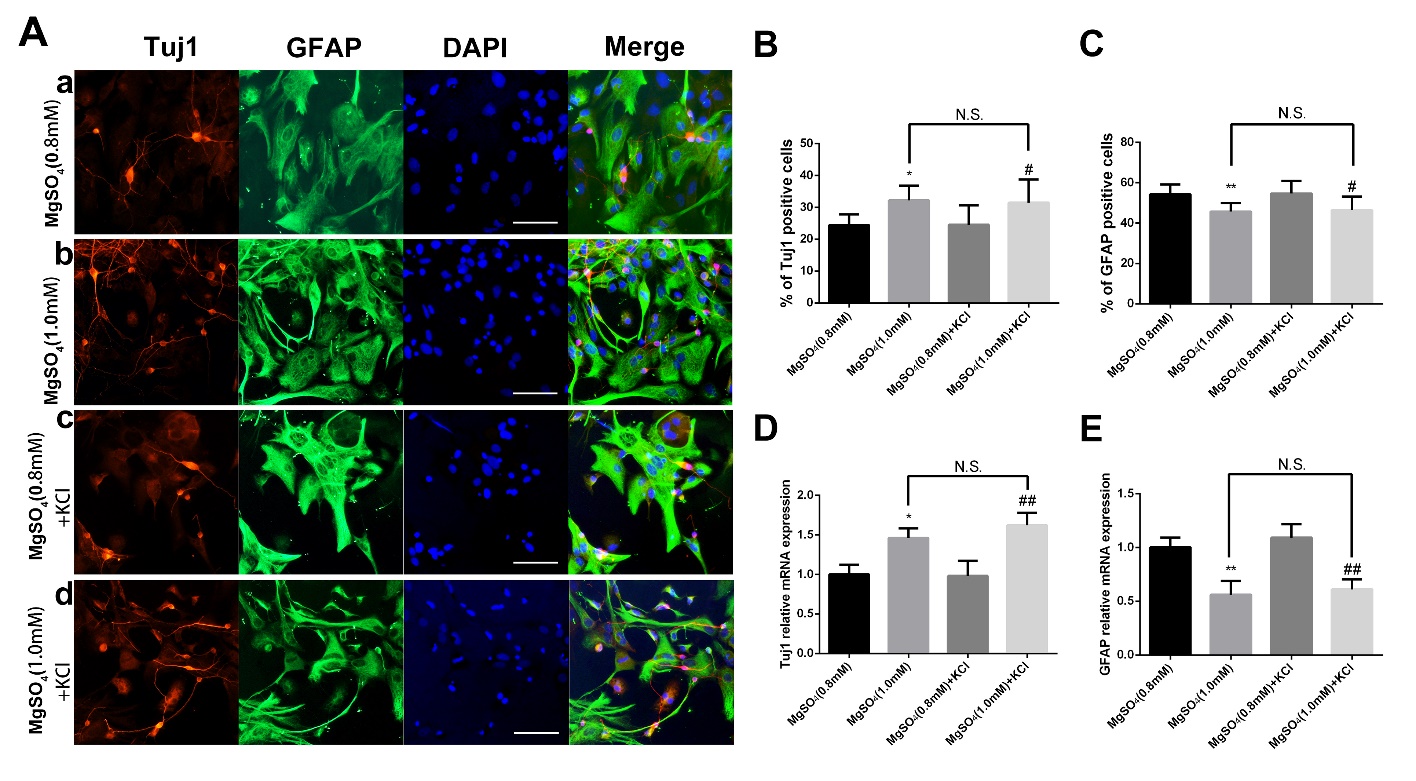


Supplementary Figure 1

Effect of KCl on the expression of Tuj1 and GFAP at elevated magnesium concentrations.

No significant change was observed when KCl (15mM) was added compared with MgSO_4_ treatment alone (*P* > 0.05).

**(A-C)** The percentage of Tuj1-positive cells increased and the percentage of GFAP-positive cells decreased (n=10).

**(D, E**) The qRT-PCR analysis of the relative mRNA expression of Tuj1 and GFAP (n=3).

N.S.: *P* > 0.05, **P* < 0.05 vs. [MgSO_4_]_0.8mM_, ***P* < 0.01 vs. [MgSO_4_]_0.8mM_; #*P* < 0.05 vs. [MgCl_2_] _0.8mM_+KCl, ##*P* < 0.01 vs. [MgCl_2_] _0.8mM_+KCl.

GFAP, glial fibrillary acidic protein; qRT-PCR, quantitative real-time polymerase chain reaction


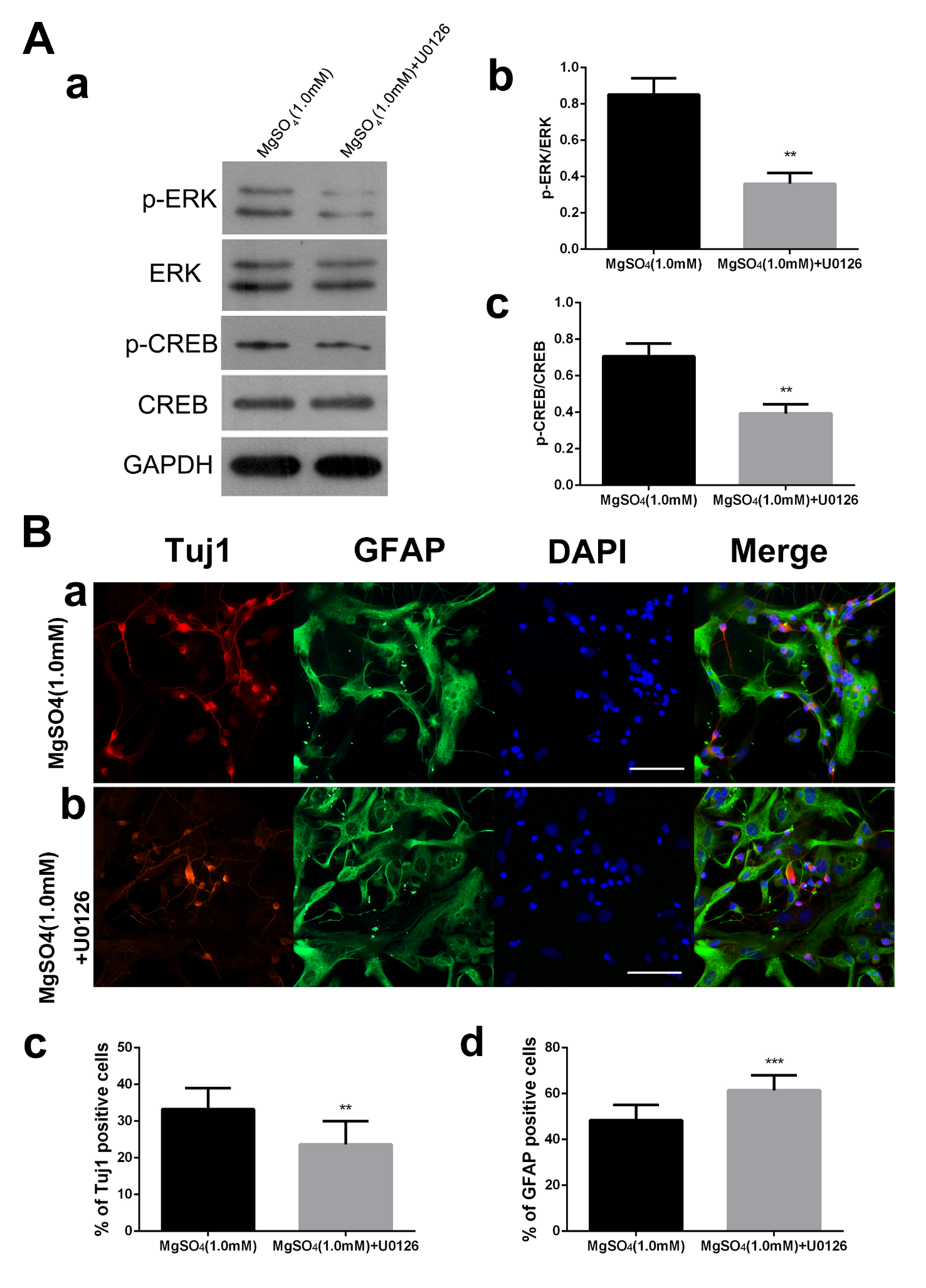


Supplementary Figure 2

**ERK/CREB activation and the expression of Tuj1 and GFAP in response to elevated magnesium and ERK inhibitor U0126.**

**(A)** Supplemented with U0126 (0.3µM), both p-ERK/ERK and p-CREB/CREB ratios decreased compared with the group with elevated magnesium concentration (1.0mM) without U0126 (*P* < 0.01) (n=3).

**(B)** Treated with U0126, the percentage of Tuj1-positive cells decreased and the percentage of GFAP-positive cells increased compared with the group with elevated magnesium concentration (1.0mM) (*P* < 0.01) (n=10).

***P* < 0.01 vs. [MgSO_4_]_1.0mM_; ****P* < 0.001 vs. [MgSO_4_]_1.0mM_; CREB, cAMP response element–binding protein; ERK, extracellular signal–regulated kinase; GFAP, glial fibrillary acidic protein.
